# Standardization of the Colombian version of the PHQ-4 in the general population

**DOI:** 10.1186/1471-244X-14-205

**Published:** 2014-07-19

**Authors:** Rüya-Daniela Kocalevent, Carolyn Finck, William Jimenez-Leal, Leon Sautier, Andreas Hinz

**Affiliations:** 1 Institute and Policlinic for Medical Psychology, University Medical Center Hamburg-Eppendorf, Hamburg, Germany; 2 Department of Psychology, Universidad de Los Andes, Bogotá, Colombia; 3 Department of Medical Psychology and Medical Sociology, University of Leipzig, Ph.-Rosenthal-Str. 55, 04103 Leipzig, Germany

**Keywords:** Depression, Standardized data, PHQ-4, General population

## Abstract

**Background:**

The PHQ-4 is a widely used open access screening instrument for depression and anxiety in different health care and community settings; however, empirical evidence of its psychometric quality in Colombia is lacking. The objectives of the current study were to generate normative data and to further investigate the construct validity and factorial structure of the PHQ-4 in the general population.

**Methods:**

A nationally representative face-to-face household survey was conducted in Colombia in 2012 (n = 1,500). The item characteristics of the PHQ-4 items, including the inter-item correlations and inter-subscale correlations, were investigated. To measure the scale’s reliability, the internal consistency (Cronbach’s α) was assessed. For factorial validity, the factor structure of the PHQ-4 was examined with confirmatory factor analysis (CFA).

**Results:**

The Cronbach’s alpha coefficient for the PHQ-4 was 0.84. The confirmatory factor analysis supported a two-factor model, which was structurally invariant between different age and gender groups. Normative data for the PHQ-4 were generated for both genders and different age levels. Women had significantly higher mean scores compared with men [1.4 (SD: 2.1) vs. 1.1 (SD: 1.9), respectively]. The results supported the discriminant validity of the PHQ-4.

**Conclusions:**

The normative data provide a framework for the interpretation and comparisons of the PHQ-4 with other populations in Colombia. The evidence supports the reliability and validity of the two-factor PHQ-4 as a measure of anxiety and depression in the general Colombian population.

## Background

Depression is one of the most common mental health disorders in community settings and a major cause of disability [1]. It is projected to be the leading cause of disease burden globally by 2030. Recently reported data on major depressive disorders in the general population yielded a 12-month prevalence of 5.7% in Europe and 6.7% in the U.S. [2,3]. The National Institute for Health and Clinical Excellence (NICE) and the recently revised American Psychiatric Association (APA) guidelines for the treatment of depression both indicate that depression should be screened and further evaluated before the initiation of treatment [4,5].

According to the World Mental Health Survey Initiative, the lifetime prevalence of a major depressive episode was 13.3% and the 12-month prevalence was 6.2% in Colombia. These values are lower than the prevalences that have been reported for the U.S. and higher than those of Europe [2,3,6]. An additional study on the prevalence of depression and related factors in Colombia reported that 10% of the study sample (N = 1,116) had a depressive episode in the past 12 months and 8.5% had a depressive episode in the past month [7]. The Third National Study of Mental Health in Colombia from 2003 reported the following prevalence rates for a major depressive disorder: lifetime prevalence = 12.1%, the last 12 months = 6.9%, and the last month = 2.1% [8]. The proportion of depression in Colombia was higher for women (Odds ratio: 1.9) than for men [6,9]. In sum, recurrent depression and depressive episodes are highly prevalent in the Colombian population. Nevertheless, the Ministerio de Salud y Protección Social (Ministry for Health and Social Protection) reported that only 14.2% of those with affective disorders received appropriate treatment (which includes not only psychiatry but also general and specialized medicine as well as social services and alternative medicine) within the last 12 months. This result demonstrates a lack of timely diagnosis and trained professionals to address both depression and anxiety disorders [8].

In Colombia, the lifetime prevalence of any anxiety disorder was reported to be 25.3% [10]. The inter-cohort differences in lifetime risk of any DSM-IV anxiety disorder yielded no higher risk in comparison to the prevalence rates of other international cohorts [10]. There is insufficient knowledge on well-validated, self-report screening instruments for the diagnostic process [11]. Although not yet included in treatment guidelines, screening for anxiety was recently suggested as a necessary first step for improving the outcomes of patients with anxiety disorders [12].

With the aim of improving the average physicians’ detection rates in the U.S. and in Germany, an ultra-brief self-report screening instrument for depression and anxiety, the 4-item Patient Health Questionnaire-4 (PHQ-4), has been developed and validated [13,14]. This instrument consists of a 2-item depression scale (PHQ-2) [15,16] and a 2-item anxiety scale (GAD-2) [17]. The bi-dimensionality of the PHQ-4 has been proven [14]. The psychometric properties and population-based norms are only available for a representative German sample [14]. However, the application of translated questionnaires in other cultures or countries may present some potential difficulties and loss of precision with regard to the comparison of norms [18].

An examination of the associations between the PHQ-4 and other health-related constructs yielded significant negative correlations with self-esteem (*r* = -.49), life-satisfaction (*r* = -.39), and resilience (*r* = -.35) [14]. Furthermore, demographic risk factors were reported for depression and anxiety. Women exhibited higher depression and anxiety scores compared to men, both scores increased with age, and subjects who lived with a partner displayed lower scores compared to subjects who lived without a partner. Moreover, the depression and anxiety scores were higher in individuals with lower educational levels and lower household incomes compared to those with higher educational levels and higher incomes. Unemployed subjects had considerably higher PHQ-4 scores compared to employed subjects [14].

To date, the screening of depression and anxiety disorders in Colombia has fallen short [8]. The main aim of the present study was to standardize the PHQ-4 in Colombia and to provide normative data for the PHQ-4 for the general population sample of different age groups and both genders. In addition, we addressed the divergent validity of the Colombian PHQ-4 with associations with self-efficacy, quality of life satisfaction, hopelessness, and emotional distress. Furthermore, we examined the demographic risk factors for depression and anxiety to provide further evidence for construct validity. Based on the results of a previous study with the PHQ-4 and according to cross-national results, we expected that women would have higher scores than men and that levels of depression and anxiety would increase with age and lower levels of education [6,14]. Furthermore, we re-investigated the two-factor structure of the PHQ-4 in the Colombian general population.

## Methods

### Study sample

The study sample included adults (18 years and above) from the general population of Colombia. A research market company (“Brandstrat Inc.”) conducted the interviews in the following eight main cities of Colombia: Bogotá, Cali, Medellín, Barranquilla, Bucaramanga, Pereira, Cartagena, and Manizales. Trained interviewers asked the eligible participants to take part in the study. If the participant consented, the interviewers asked them to complete a booklet with several questions and questionnaires. After the participants completed the booklet, the interviewers checked it for missing data. If data were missing, the interviewers asked the participants to fully complete the questionnaires (except household income). Each Colombian city is divided into barrios (quarters) with different mean socioeconomic strata (SES) of the inhabitants (SES ranging from 1 = very low to 6 = very high). The sampling procedure that was adopted in this survey assured that each stratum (with corresponding barrios) was representatively included in the sample. Within each barrio, the participants were randomly selected. In the case of non-response, another eligible participant from the same stratum was asked to participate. This technique yielded a stratum distribution in the study sample that is identical with that of the general population. Due to this procedure, the resulting sample can be assumed to be representative of the population of Colombia living in private houses. A total of 2,372 individuals were contacted, of which 1,500 responded with complete data sets, resulting in a response rate of 63%. The interviewers did not obtain data in the case of non-participation. Therefore, we have no data on the reasons for non-participation. The total duration needed to complete the questionnaires was approximately 45 min. As an incentive to collaborate in the study, the participants were provided with a brochure with information about healthy lifestyles. The Ethics Committee at the Universidad de los Andes approved the study, and informed consent was obtained from all participants.

### Measures

#### *PHQ-4*

The PHQ-4 consists of two validated ultra-brief screeners for depression and anxiety [13,14]. Each of the items corresponds to the DSM-IV Diagnostic Criterion A symptoms for major depressive disorder and generalized anxiety disorder, respectively [19]. The response options are “not at all”, “several days”, “more than half the days”, and “nearly every day”, which are scored as 0, 1, 2 and 3, respectively. The PHQ-4 scores range from 0 to 12 [13].

To assess the construct validity of the PHQ-4, the survey also included the following questionnaires on emotional distress, hopelessness, life satisfaction, general health, and self-efficacy:

#### *HADS*

The Hospital Anxiety and Depression Scale consists of 14 items, seven items that indicate anxiety and seven items that indicate depression. The answer format offers four options that are scored from 0 to 3. This results in values that are between 0 and 21 for each scale [20].

#### *DT*

The Distress thermometer is a single-item, self-report measure of psychological distress [21]. This visual-analogue scale has scores from 0 ‘no distress’ to 10 ‘extreme distress’. Using the scale, the participants were asked to rate how distressed they felt in the past week.

#### *BHS*

The Beck Hopelessness Scale was also used [22]. The 20 dichotomized questions of the instrument measure positive and negative attitudes about the future. Higher scores on this scale indicate higher levels of hopelessness.

#### *QLS*

The Questions on Life Satisfaction assesses general life satisfaction in the following eight dimensions: friends/acquaintances, leisure activities/hobbies, health, income/financial security, occupation/work, housing/living conditions, family life/children, and partner relationship/sexuality [23]. In addition, the subjective importance of each of the dimensions is assessed. Finally, the total QLS score is calculated as the sum of the satisfaction scores of the eight dimensions, weighted by their importance ratings.

#### *GHQ-12*

The 12-item General Health Questionnaire is a validated indicator of psychological distress [24]. In this study, we used the one-dimensional Likert scaling (0–1–2–3). The points were summed to a global score that ranged from 0 to 36.

#### *GSES*

The General Self-Efficacy Scale, developed by Schwarzer and Jerusalem (1995), was used to assess the participants’ subjective evaluation of their ability to cope with and solve problems and demands [25]. Ten items are answered on a four-point scale, with higher sum scores indicating higher self-efficacy.

### Data analysis

The item characteristics of the PHQ-4 items, including item inter-correlations, were calculated. Concerning reliability, the internal consistency of the PHQ-4 was assessed. The factor structure was tested with confirmatory factor analysis (CFA), using the maximum likelihood approach. The model fit of the CFA was tested using the following fit indices: the minimum discrepancy divided by its degrees of freedom (CMIN/DF); the goodness-of-fit-index (GFI); the normed-fit-index (NFI); the Tucker-Lewis-Index (TLI); the comparative-fit-index (CFI); and the root mean square error of approximation (RMSEA). For a good model fit, the ratio CMIN/DF should be close to 3 or smaller [26]. Yet, there are several shortcomings with the χ^2^ statistics, such as its dependence on the sample size. With increasing sample size and a constant number of degrees of freedom, the χ^2^ value increases. This leads to the problem that plausible models might be rejected based on a significant χ^2^ statistic even though the discrepancy between the sample and the model-implied covariance matrix is irrelevant. Yet, the analysis of covariance structures is grounded in large-sample theory. As such, large samples are critical to obtaining precise parameter estimates.

Therefore, limited emphasis should be placed on the significance of the χ^2^ statistic. Jöreskog and Sörbom (1993) suggested the use of χ^2^ not as a formal test statistic but, rather, as a descriptive goodness-of-fit index.

Furthermore, GFI, NFI, TLI, and CFI values that are higher than 0.90 indicate an acceptable model fit. The RMSEA values should be <0.10 [26,27]. Additional analyses were conducted to test the invariance of the model across both gender and different age groups using multi-group CFA. This is an important statistical condition before the means of different subgroups can be compared with each other [28]. The measurement invariance was tested in three steps using the configural, combined model (no constraints), followed by a metric invariant model (with equal item loadings, that is, the paths and covariances were constrained to be equal), and a scalar invariant model (with equal item loadings and item intercepts across groups) [29]. Because these models are hierarchically nested and increasingly restricted, the models were then compared to each other on the basis of the ΔCFI. Values ≤ .01 indicate the invariance of the model [30]. Invariance tests have proven themselves as a necessary step in group analyses (e.g. gender, age, cross-culture).

We investigated the PHQ-4 scale correlations with the HADS [20], the Distress Thermometer [21], the Beck Hopelessness Scale [22], the Questions on Life Satisfaction [23], General Health Scale [24], and General Self-Efficacy Scale [25]. In addition, we investigated group differences in sociodemographic characteristics using the χ^2^-test and Kruskal-Wallis test. The effect sizes of the subgroups for each variable with the highest and lowest mean scores were considered when calculating Cohen’s *d*, which represents the difference between the means divided by the standard deviation [31]. Additionally, η^2^ was used as a measure of effect size for use in ANOVA. Effect sizes were defined as follows: “small, d = .2, η^2^ = .02”, “medium, d = .5, η^2^ = .13”, “large, d = .8, η^2^ = .26” [32,33].

The percentiles were calculated according to the following formula [34]: percentile rank = 100* (m + 0.5 k)/N, where m is the number of members of the sample who obtained a score that was lower than the score of interest, k is the number who obtained the score of interest, and N is the overall normative sample size. The statistical analyses were conducted using SPSS-19 and AMOS 20.

## Results

### Sample characteristics

The sociodemographic characteristics of the final sample are provided in Table 1. The sample is representative of the adult Colombian population in terms of age, gender, and civil status, according to data of the Departamento Administrativo Nacional de Estadística (DANE, Colombian Statistical Administrative Office) [35]. With the exception of household income, there were no missing data because the interviewers controlled the completeness of the questionnaires. The associations of the PHQ-4 scores with the demographic characteristics are shown in Table 1.

**Table 1:** Demographic characteristics of the study sample and associations with PHQ-4 scores

		**Total sample**		**PHQ-4**		**Group differences**	
		**n**	**%**	**M**	**SD**	***P *** **value**	**Effect size**^**b**^
Gender	Male	724	48.3	1.13	1.90	< .01	d = 0.14
	Female	776	51.7	1.41	2.09		
Age group, yr.	18-24	279	18.6	1.18	2.09	.16	η^2^ = 0.06
	25-34	282	18.8	1.11	1.80		
	35-44	286	19.1	1.17	1.88		
	45-54	267	17.8	1.37	2.19		
	55-64	216	14.4	1.40	1.98		
	≥ 65	170	11.3	1.55	2.11		
Cohabitation	Yes	764	50.9	1.32	2.02	.41	d = 0.04
	No	736	49.1	1.23	1.99		
Marital Status	Married	490	32.7	1.14	1.94	.10	η^2^ = 0.03
	Living together	274	18.3	1.40	2.08		
	Single	510	34.0	1.25	2.06		
	Divorced	152	10.1	1.35	1.88		
	Widowed	74	4.9	1.74	2.01		
Education	≤ 5 years	228	15.2	1.77	2.26	<.001	η^2^ = 0.20
	6-9 years	544	36.3	1.38	2.04		
	10-13 years	266	17.8	1.12	2.00		
	≥ 14 years	431	9.6	1.03	1.76		
	None	31	2.1	1.52	2.13		
Employment	Pupil or Student	183	12.2	1.34	2.19	<.01	η^2^ = 0.12
	Unemployed	107	7.1	1.36	2.03		
	Handicapped	21	1.4	2.71	3.29		
	Retired	111	7.4	1.41	1.87		
	Housewife/Househusband	348	23.2	1.41	1.96		
	Employed	730	48.7	1.12	2.93		
Net household income^a^	< 400,000	145	11.5	1.78	2.45	< .001	η^2^ = 0.02
	400,000 to 800,000	403	32.0	1.29	1.89		
	800,000 to 1600,000	317	25.2	1.34	2.03		
	> 1600,000	393	31.2	0.97	1.76		

^a^Income in Colombian pesos (COP), 1,000 COP = 0.43 EUR = 0.57 USD.

^b^Effect sizes defined as follows: “small, d = .2, η^2^ = .02”, “medium, d = .5, η^2^ = .13”, “large, d = .8, η^2^ = .26”.

There were significant effects of gender, educational level, employment status, and income in the Colombian general population. As noted in Table 1, the calculated effect sizes were low for gender (*d* = .14) and household income (η^2^ = .02) and moderate for employment (η^2^ = .12) and large for education (η^2^ = .20).

### Internal consistency

The internal consistency (Cronbach’s α) of the PHQ-4 scale reached the value of α = 0.84. The inter-correlations of the items from the same subscale are displayed in Table 2.

**Table 2:** Inter-item correlations of the PHQ-4 and inter-subscale correlations of the PHQ-4

**Correlation matrix of items**					**Correlation matrix of subscales**			
**Item**	**1**^**a**^	**2**^**a**^	**3**^**b**^	**4**^**b**^	**Subscale**	**Total score**	**Anxiety**	**Depression**
2	.63***	1			Total score	1		
3	.44***	.59***	1		Anxiety	.91***	1	
4	.54***	.61***	.68***	1	Depression	.91***	.66***	1

^a^PHQ-2 depression item.

^b^GAD-2 anxiety item.

***p<0.001.

### Confirmatory factor analysis

The two-dimensional structure of the PHQ-4 was tested using CFA with N = 1,500 participants. All but one (RMSEA) fit index indicated a very good model fit (CMIN/DF = 32.31; GFI = 0.989; NFI = 0.987; TLI = 0.923; CFI = 0.987, RMSEA = 0.145). The standardized factor loadings ranged between 0.70 and 0.85. We also tested for a one-factor model, which yielded less favorable fit indices (CMIN/DF = 114.45; GFI = 0.964; NFI = 0.953; TLI = 0.861, CFI = 0.954, RMSEA = 0.194), with factor loadings between 0.67 and 0.82.

In the following section, we tested the invariance of the model across gender and age (see Table 3). The age groups were defined according to [14] for reasons of comparability. Thus, the total sample was split into a younger group (≤48 years) and an older group (>48 years). The results indicated that the two-factor model was structurally invariant between age and gender groups. The values of ΔCFI were smaller than 0.01 indicating that the null hypothesis of invariance should not be rejected [30].

**Table 3:** Test for invariance across gender and age using multi-group CFA

	**χ**^**2 **^**(df)**	**Δχ**^**2**^	**ΔP**	**CMIN/df**	**CFI**	**Δ CFI**	**RMSEA**^**1**^	**ΔRMSEA**
**Gender**								
Men	20.68 (1)			20.68	.98		.165	
Women	11.49 (1)			11.497	.99		.116	
**Multigroup analysis**								
Configural model	32.18 (2)			16.09	.98		.100	
Metric model	33.76 (4)	1.58	<.001	8.44	.98	.000	.070	.03
Scalar model	51.43 (8)	19.25	<.001	6.42	.98	.005	.060	.04
**Age**								
≤48 years	9.55 (1)			9.55	.99		.106	
>48 years	21.01 (1)			21.01	.98		.165	
**Multigroup analysis**								
Configural model	30.56 (2)			15.28	.98		.098	
Metric model	33.37 (4)	2.81	<.001	8.34	.98	.000	.070	.02
Scalar model	49.89 (8)	19.33	<.001	6.23	.98	.005	.059	.03

^1^Models with fewer items and factors are associated with larger standard errors in RMSEA [36].

### Construct validity

The correlations between the PHQ-4 total score and the Hospital Anxiety and Depression Scale [20], the Distress Thermometer [21], the Beck Hopelessness Scale [22], the Questions on Life Satisfaction [23], the General Health Questionnaire [24], and the General Self-Efficacy Scale [25] are summarized in Table 4. The correlations with the PHQ-4 were highest for the total score of the Hospital Anxiety and Depression Scale (*r* = 0.46, p < 0.001) and the General Health Questionnaire (*r* = 0.44, p < 0.001), indicating convergent validity. Divergent validity can be assumed in terms of the low correlations of the PHQ-4 with self-efficacy (*r* = -0.26, p < 0.001) and life satisfaction (*r* = -0.29, p < 0.001).

**Table 4:** Correlations between the PHQ-4 subscales and concurrent validity measures

	**PHQ-4**	**PHQ-4 subscales**	
		**Anxiety**	**Depression**
Anxiety and depression (HADS)			
Total Score	.46***	.37***	.47***
Anxiety	.45***	.39***	.44***
Depression	.40***	.30***	.42***
Hopelessness (BHS)			
Total Score	.27***	.21***	.28***
General mental health (GHQ-12)			
Total Score	.44***	.37***	.42***
Self-efficacy (GSES)			
Total Score	-.26***	-.21***	-.26***
Distress (DT)			
Total Score	.41***	.40***	.35***
Life satisfaction (QLS)			
Total Score	-.29***	-.25***	-.27***

***p < .001.

### Normative data

The normative data for the PHQ-4 were generated for both genders (51.7% female) and different age levels (mean age (SD) of 41.8 (16.2) years). Table 5 summarizes the normative data for the different age levels and both genders. The percentiles from this table can be used to compare an individual subject’s PHQ-4 score with those that were determined from the Colombian general population reference group based on age and gender. For example, a PHQ-4 score of 4 for a 36-year-old man indicates a percentile rank of 89.1% in the total population and 91.7% in a group of subjects of the same age and gender. Likewise, a PHQ-4 score of 4 for a 36-year-old woman corresponds to a percentile rank of 89.1% in the total population and 88.6% in the same age and gender group.

**Table 5:** Normative data from the general population for the PHQ-4

	**Total**	**Men**						**Women**					
	**18-90 y.**	**18-24**	**25-34**	**35-44**	**45-54**	**55-64**	**≥65**	**18-24**	**25-34**	**35-44**	**45-54**	**55-64**	**≥65**
	**N = 1500**	**n = 147**	**n = 128**	**n = 132**	**n = 118**	**n = 108**	**n = 91**	**n = 132**	**n = 154**	**n = 154**	**n = 149**	**n = 108**	**n = 79**
M	1.27	1.01	0.98	1.02	1.36	1.25	1.22	1.37	1.21	1.31	1.38	1.56	1.94
S.D.	2.01	1.81	1.76	1.84	2.13	1.95	1.95	2.36	1.84	1.90	2.23	2.01	2.23
Sum Score	Percentile^a^												
0	28.3	33.1	30.9	31.8	28.8	28.2	28.6	29.9	28.6	27.9	28.9	21.3	22.2
1	63.6	71.3	70.7	71.6	63.1	64.8	64.3	64.4	62.0	62.3	64.4	51.4	50.0
2	75.0	80.4	83.6	81.4	73.3	76.4	76.9	74.6	73.4	73.1	75.5	69.4	60.8
3	82.9	86.8	89.1	86.4	80.9	82.9	85.2	83.0	84.1	81.8	81.9	82.9	69.6
4	89.1	91.2	92.6	91.7	88.6	89.8	31.2	88.6	91.9	88.6	86.2	89.8	80.4
5	93.1	94.3	94.5	94.3	93.2	94.0	95.1	92.8	96.4	92.9	90.3	94.0	89.2
6	95.6	96.6	96.5	96.2	94.9	95.8	96.7	95.1	98.4	96.8	93.6	94.0	94.3
7	97.6	98.7	98.4	98.1	97.5	97.7	96.7	96.6	99.4	99.3	97.0	97.2	97.5
8	98.9	100.0	99.6	99.6	99.2	99.5	98.4	97.7	99.4	100.0	99.3	97.2	99.4
9	99.5	100.0	99.6	99.6	99.2	99.5	100.0	97.7	99.4	100.0	99.3	99.1	99.4
10	99.6	100.0	99.6	99.6	99.2	99.5	100.0	98.5	99.4	100.0	99.3	100.0	99.4
11	99.7	100.0	99.6	99.6	100.0	99.5	100.0	98.5	99.4	100.0	99.3	100.0	99.4
12	99.9	100.0	99.6	99.6	100.0	99.5	100.0	99.6	100.0	100.0	100.0	100.0	99.4

^a^Rank of the subject compared to other subjects of the same age group and gender.

## Discussion

A main result of this study was the standardization of the PHQ-4 in Colombia with the provision of normative data from the general population. Given that age- and gender-specific comparative data were generated based on subgroups that consisted of 73 to 180 subjects each, the sample sizes were sufficient to provide sound normative data. These norms can be used to compare a subject’s scale score with those that were determined from a general population reference group [37,38]. Although normative data of the PHQ-4 in the German general population were previously available [14], this study is the first to provide normative data for the Colombian general population.

The PHQ-4 means were lower in Colombia compared to the German sample [1.27 (SD: 2.01) vs. 1.76 (2.06), respectively]. The previous analyses of the PHQ-4 factor structure yielded two subscales, anxiety and depression [13,14]. This factor structure was confirmed in the current study. The confirmatory factor analysis supported a two-factor model, which was structurally invariant between different age and gender groups. These results are similar to those in the German general population, in which all of the tested models were structurally invariant between different age and gender groups [14].

The present study, including 1,500 subjects, provides evidence that the PHQ-4 is a reliable and valid ultra-brief self-report measure in the general population. Specifically, the correlations of the PHQ-4 with life satisfaction (*r* = -0.29) are similar to the correlations between these scales in previous studies, supporting the construct validity of the PHQ-4 [14,39]. In the original PHQ-4 validation study, which comprised 2,149 unselected primary care patients, higher PHQ-4 scores were strongly associated with worse functioning on all six SF-20 scales (a questionnaire on quality of life) and increased disability days and health care utilization [13]. The differences in correlations with the HADS and GHQ compared to the other scales were moderately larger. Interventions aimed at early detection and treatment might help to reduce the persistence or severity of primary anxiety and depressive disorders and prevent the onset of secondary disorders. A review of randomized controlled trials with the implementation of screening for depression symptoms in routine care revealed little or no impact on the recognition, management, or outcome of depression in primary care or the general hospital [40]. However, a web-based self-screening and secure communication system was evaluated at the University of Washington for 17 months. Of the subjects who used the system, 75% noted that the system helped them to make a decision to receive help from professionals [41].

Some limitations of the current study should be mentioned. Due to the cross-sectional design of this study, it was not possible to calculate the test-retest reliability of the PHQ-4. A further limitation of this general population study is that it did not include standard criterion interviews, which would have allowed for the calculation of specificity and sensitivity for the optimal cut point and construction of a receiver operating characteristic (ROC) curve. For the PHQ-2 and the GAD-2, scale scores of ≥3 were suggested as cut-off points between the normal range and probable cases of depression or anxiety [15,16,42,43]. These cut-off points were based on the receiver operating characteristic (ROC) analyses that were conducted in previous primary care validation studies [17]. The response rate of 63% indicates that nearly one-third of the contacted individuals did not participate. In the case of non-response, another eligible participant from the same stratum was recruited. However, it is possible that the sample has some selection bias.

In general, reducing the burden and enhancing the early detection of mental disorders require major shifts in research, clinical practice, and public health by incorporating multidisciplinary models of intervention. Such changes have begun in the U.S. (see http://www.nihpromis.org) and the European Union (see http://www.roamer-mh.org); however, in Latin America, these changes are a task of the future.

## Conclusions

Depressive and anxiety syndromes are a common problem in health care services and are associated with substantial functional impairment and health care utilization. Thus, valid screening is necessary in health care and community settings. The PHQ-4 is a good tool for this task. Normative data for the PHQ-4 in the Colombian general population were provided and can be used for interpretation and comparisons with other populations.

## Competing interests

The authors declare that they have no competing interests.

## Authors’ contributions

AH was the principal investigator and was responsible for the study design. CF and WJ collected the data and were responsible for data accuracy. RK wrote the manuscript. LS and RD performed the statistics. AH and CF commented on the first draft of the manuscript. All authors read and approved the final manuscript.

## Pre-publication history

The pre-publication history for this paper can be accessed here:

http://www.biomedcentral.com/1471-244X/14/205/prepub
